# **“**What are we doing to our babies’ teeth?” Barriers to establishing oral health practices for Indigenous children in South Australia

**DOI:** 10.1186/s12903-021-01791-x

**Published:** 2021-09-06

**Authors:** Brianna Poirier, Joanne Hedges, Lisa Smithers, Megan Moskos, Lisa Jamieson

**Affiliations:** 1 Australian Research Centre for Population Oral Health, Adelaide Dental School, University of Adelaide, Adelaide, 5000 Australia; 2 School of Public Health and the Robinson Research Institute, University of Adelaide, Adelaide, 5000 Australia; 3 The School of Health and Society, University of Wollongong, Wollongong, 2522 Australia; 4 Future of Employment and Skills Research Centre, Faculty of the Professions, University of Adelaide, Adelaide, 5000 Australia

**Keywords:** Indigenous health, Oral health, Early childhood caries, Indigenous oral health, Nutrition knowledge, Oral health knowledge, Dental public health, Social determinants of health

## Abstract

**Background:**

During the 1970s, optimal oral health was experienced more frequently amongst Indigenous children in Australia than their non-Indigenous counterparts. As a result of public health interventions targeting oral disease, oral health has improved for most children; however, Indigenous children today experience oral disease at alarmingly high rates. A history of colonisation, assimilation, racism and cultural annihilation has had profound impacts on oral health for Indigenous peoples; compounded by environmental dispossession and a shift from traditional diets to one of processed and nutrient-poor foods, often high in sugar.

**Methods:**

This project aimed to identify factors related to the increased occurrence of caries in Indigenous children. Using purposive sampling from the larger project, this paper thematically analyses 327 motivational interviews to explore current barriers impeding parental efforts to establish oral health and nutrition practices for Indigenous children. Representation of socioeconomic positions of families were compared across themes, as based on maternal age, employment, residency and number of children in care.

**Results:**

Findings resulted in a conceptual model of barriers that exist across knowledge, social, structural and parental factors. Major thematic results include: social consumption of processed foods, busy households, misleading nutrition marketing, sugar cravings and lack of oral health and nutrition knowledge.

**Conclusion:**

A discussion of the findings results in the following recommendations increased oral health promotion efforts in non-metropolitan areas; utilisation of community experiences in creating strategies that encourage oral health and nutrition knowledge; and the extension of oral health initiatives and future research to include all family members.

*Trial registration* Australian New Zealand Clinical Trial Registry ACTRN12611000111976; registered 01/02/2011.

**Supplementary Information:**

The online version contains supplementary material available at 10.1186/s12903-021-01791-x.

## Background

According to the United Nations, Indigenous peoples include all those “having a historical continuity with pre-invasion and pre-colonial societies that developed on their territories, who consider themselves distinct from other sectors of the societies now prevailing on those territories” [1]. Globally, Indigenous peoples experience a disproportionate burden of disease for many conditions, including obesity, non-insulin dependent diabetes, and dental caries [2]. A history of colonisation, government-enforced assimilation, racism and cultural annihilation has had profound impacts on Indigenous health and is reflected in health inequities sustained by Indigenous communities today [3–5]. The forcible removal of communities from traditional lands, loss of traditional customs and languages, and subsequent environmental dispossession is an additional contributing factor to poor health because it has resulted in a transition from nutrient-dense traditional foods, to processed, nutrient-poor Western foods that are high in sugar [6, 7].

In Australia, Aboriginal and/or Torres Strait Islander (respectfully, subsequently referred to as ‘Indigenous’) communities flourished for 65,000 years prior to European invasion and colonisation [8]. Economic and social discrimination, processed diets, infectious disease, environmental dispossession and child removal are some of the ways in which processes of colonisation and government policies have intentionally disrupted Indigenous health in Australia [9]. Despite the Australian government’s considerable resources allocated to addressing health inequities between Indigenous and non-Indigenous Australians, disparities continue to escalate [10, 11]. In Australia, 61% of Indigenous children experience decay in their primary teeth compared to 41% of non-Indigenous children and Indigenous children are more likely to have untreated decay in at least one primary tooth (44%) than non-Indigenous children (26%) [12].

The deleterious impacts of poor oral health in children are well documented. Pain, speech difficulties, lowered self-esteem and difficulty eating or sleeping are common consequences of ECC [13]; evidence suggests that more severe consequences impact children’s growth, development, concentration, education attainment, quality of life, failure to thrive and can be life-threatening in some cases [14–18]. While ECC can have serious ramifications on health, the disease is entirely preventable with limited sugar consumption, proper oral hygiene, regular dental visits and sufficient fluoride exposure [18–20]. Childhood dental disease is the strongest indicator for adult dental disease [16, 21] and the greatest impact on childhood oral hygiene practices is caregiver influence, underscoring the importance of prevention efforts aimed at young children within the family setting [18, 22, 23]. Varying degrees of success have been experienced with population-level interventions for oral health, with water fluoridation being one of the most successful interventions in reducing ECC to date [22, 24]. Despite fluoridation and educational programs for children and parents, barriers to oral health prevention persist for Indigenous communities as evidenced by the prevalence of ECC among Indigenous children [25].

In 2007, an Australian public service report detailed Indigenous health as a ‘wicked’ problem, difficult to solve and symptomatic of deeper concerns [26]. Present prevention strategies and policies do not consider the impact of issues, such as colonisation or structural barriers, that Indigenous peoples face in establishing good oral health [9, 27]. Developing contextual understandings of the environments in which these health inequities persist is necessary when addressing such vast disparities [28]. Qualitative research offers an opportunity to further explore the experience and context of poor oral health among Indigenous peoples that has been extensively documented by quantitative findings. Therefore, the aim of this paper is to explore the complex context in which Indigenous Australians experience oral health, collate and interpret participants’ experiences and develop an understanding of current barriers impeding parental efforts to establish oral health practices for their Indigenous children.

## Methods

### Method

Motivational Interviewing (MI) is a psychotherapy intervention that encourages participants to identify, explore and resolve obstacles to behaviour change [29]. Contrary to traditional health education approaches, MI is an empathetic behavioural support method rooted in the notion that knowledge alone is insufficient to elicit behaviour change, and that intrinsic motivation increases likelihood of behaviour change. MI creates an exploratory atmosphere for participants to articulate personal values, capacities and motives for behaviour change; emphasising an individual’s personal motivation for change [30]. MI has previously been used to elicit oral health behaviour change for parents and their children [31, 32]. Importantly, MI parallels cultural values of Indigenous peoples, including oral traditions of storytelling and yarning [33], respects self-determination and is better able to yield a holistic and contextual understanding of a given issue [34, 35].

### Design

This project was nested within a randomised control trial of an ECC intervention designed and conducted in partnership with Indigenous families and communities in South Australia. The protocol [36], primary quantitative results [37], and cohort profile have been published [38]. At baseline, the trial enrolled 448 women pregnant with an Indigenous child across South Australia. Participants were randomly allocated to intervention or control (delayed intervention) groups. There were four components to the intervention, (1) provision of dental care to mothers during pregnancy; (2) application of fluoride varnish to the teeth of children; (3) anticipatory guidance; and (4) MI. The findings presented in this paper are derived from the MI element of the trial. Motivational interviews were conducted with participants in the intervention group at baseline during pregnancy and when the child was aged 6-, 12-, and 18 months. The respective directives for each session were (1) encouraging dental care during pregnancy; (2) emphasising the importance of non-cariogenic foods and drinks for children; (3) emphasising the importance of fluoride in ECC prevention; (4) encouraging first dental appointment. Participants in the control group received MI at 24-, 30-, and 36 months, with the first session combining directives one and two.

### Participants and sampling

For this qualitative analysis, we utilised purposive sampling of motivational interviews, based on the fidelity scores of trained staff who conducted the MI. Fidelity is defined as the extent to which an intervention is performed as intended [39]. Fidelity assessment of MI was completed to ensure sound methodological approach and scientific rigour in this trial [40]. The success of MI is contingent on interventionist competency and fidelity in eliciting participant statements of self-motivation and resistance to change [41]. Four trained staff conducted motivational interviews with varying compliance to the MI approach and different degrees of participant engagement. All included interviews for this analysis were completed by the single staff member that had the highest MI fidelity score. This decision was made because these interviews provided the richest data, constituted the majority of collected data and interviews were more comparable with one another than across interviews by other staff, which facilitated analysis. The staff with the highest fidelity score is a senior Indigenous researcher who utilised colloquial language and established trusting relationships with participants.

### Analysis

It is important to acknowledge the assumptions one brings to qualitative research as they inescapably impact the interpretation of data and production of findings [42]. As a non-Indigenous researcher from Canada, the primary author took steps to familiarise herself with the data and the context in which it was collected prior to analysis. Local contextual and cultural understandings were enhanced through field work with the same communities and Indigenous health workers involved with this trial. Approximately one year was taken in reviewing, reading and listening to interviews. Understandings of data were extensively discussed with the senior Indigenous researcher who conducted the interviews (JH) and the project’s primary investigator (LJ) prior to initiating analysis. Braun and Clarke’s [42–44] framework for reflexive thematic analysis guided the analytic process. Reflexive thematic analysis embraces the unique subjective skills a researcher brings to the project and enables organic identification of themes [42]. Inductive themes, grounded in the data, were coded line by line with NVivo 12 software (QSR International Pty Ltd. Version 12.6.1) and without a structured codebook to provide space for engaged interpretation of data. Once all transcripts had been coded, the data was re-visited, and similar codes were aggregated for iterative thematic development. Typically, reflexive thematic analysis does not utilise summary topics in conceptual models [42], however due to the quantity of codes, themes and transcripts analysed, as well as the multi-faceted context of Indigenous oral health, they are employed here to make sense of the ways in which barriers exist for participants.

**Fig. 1:**
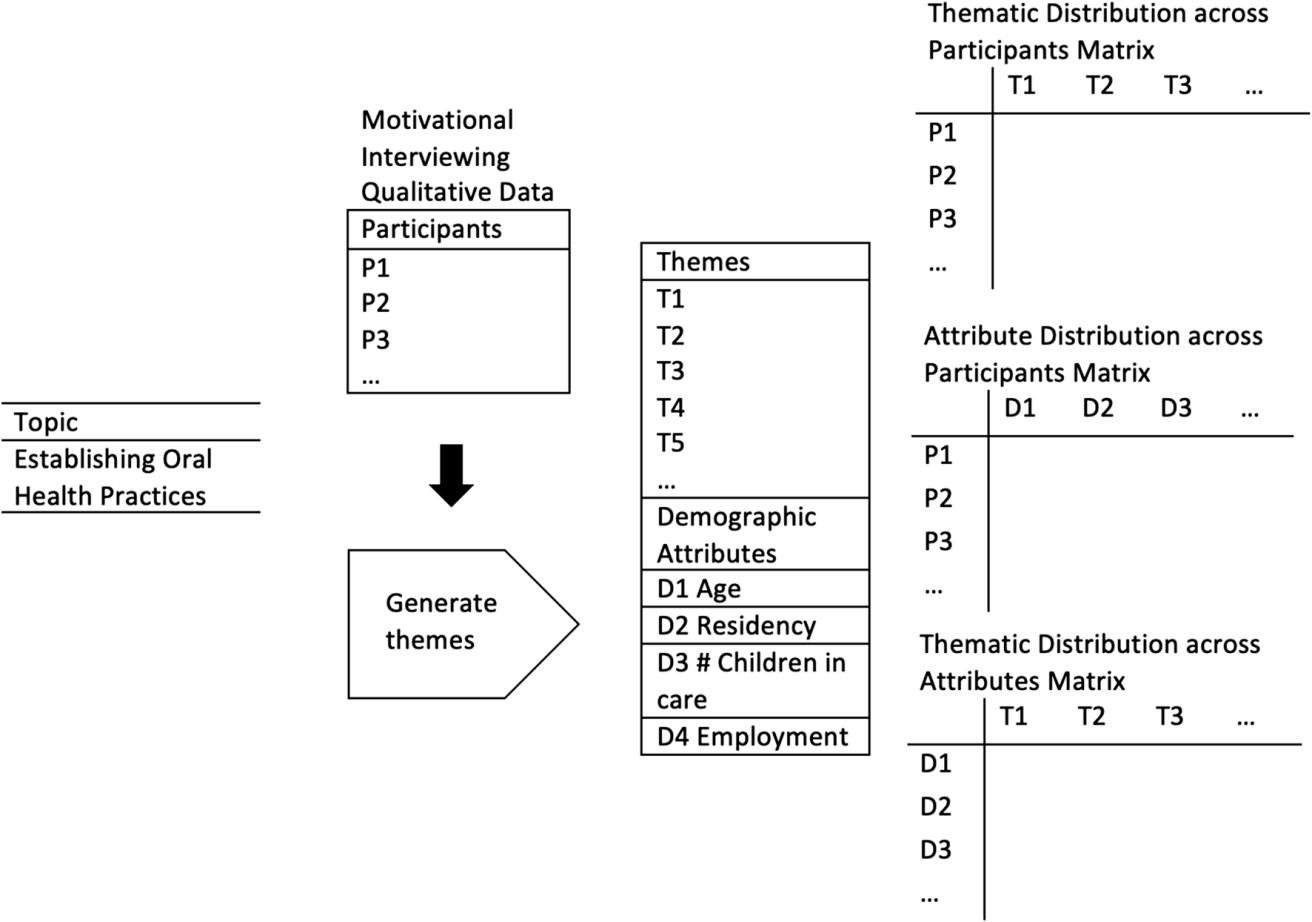
Subgroup analysis utilising matrices to compare relationships between participants, themes and demographic attributes

The data from 357 interviews and 227 participants provided a unique opportunity to explore how socioeconomic positions might contribute to oral health experiences for carers. Subgroup comparisons were based on maternal age, residential location, number of children in care and employment status. These characteristics were chosen because the Australian Institute of Health and Welfare estimates that 34% of the health gap between Indigenous and non-Indigenous Australians is attributable to social determinants of health including income, employment and overcrowding [45] and in 2018, Indigenous mothers in Australia were most likely to be aged between 20 and 24 (31%) [46]. The NVivo software attribute feature was utilised to assign characteristics to participant transcripts and thematic codes. Matrices were utilised to compare the relationships between participants and themes, demographics and participants, and demographics and themes. Subsequent analysis determined how many participants within each demographic subgroup discussed a given theme (Fig. 1).

## Results

**Fig. 2:**
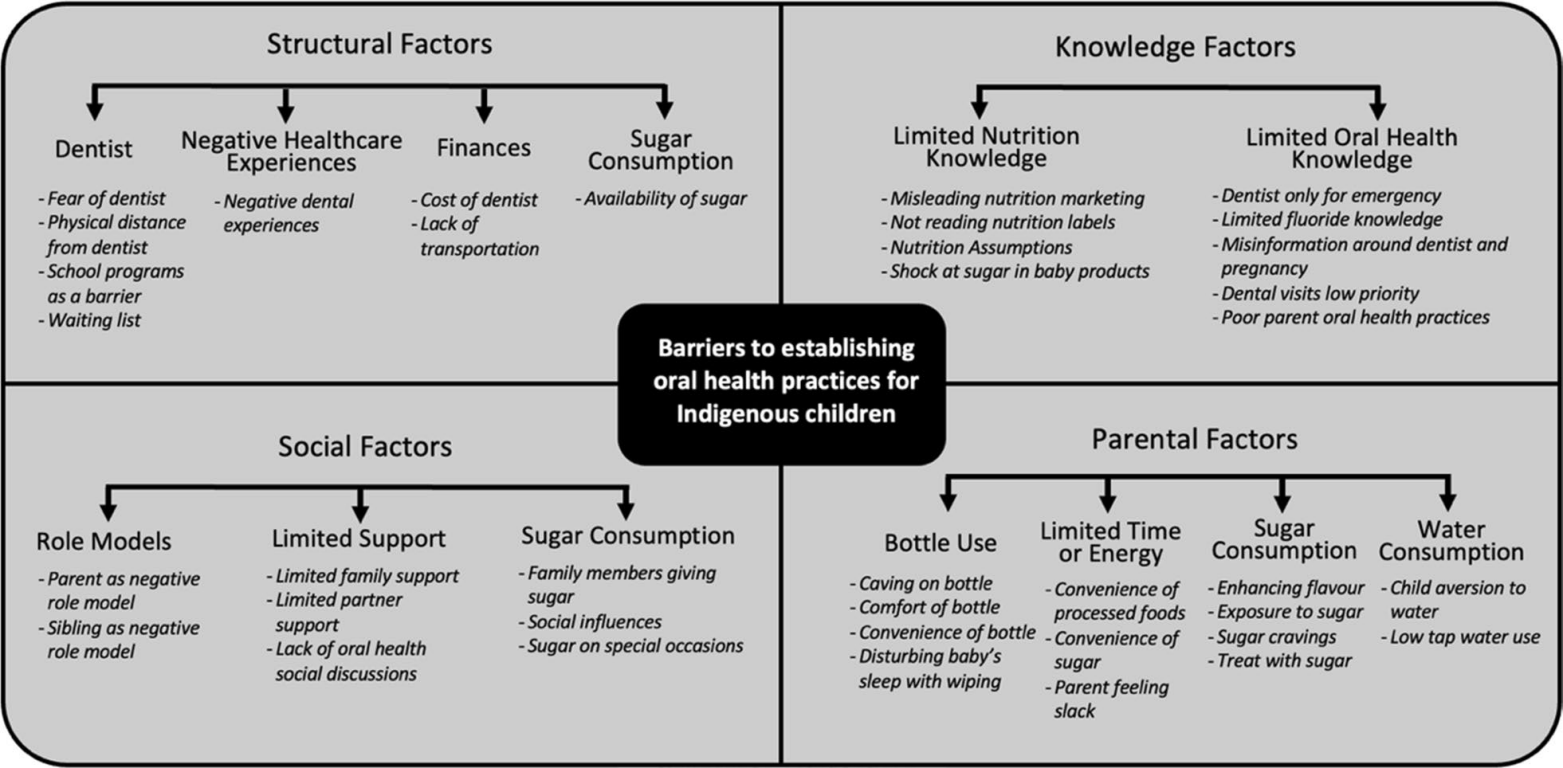
Conceptual model of barriers to establishing oral health practices for Indigenous children in South Australia

Respondents discussed a number of factors that create barriers for participants to establish oral health practices. These included knowledge factors, parental factors, structural factors and social factors (Fig. 2). The findings below are presented in order of highest to lowest frequency that respondents mentioned a particular theme: knowledge factors (3232), parental factors (1632), structural factors (902), and social factors (623). Findings represent discussions from 357 interviews with 227 parents or carers of Indigenous children aged 6–36 months from across South Australia (50.7% of baseline sample). Participant characteristics varied across the included demographic measures of employment, number of children in care, residential location and maternal age (Table 1). The majority of the sample were older than 25 (66.1%), had 1–3 children in their care (67.6%), were unemployed (69.6%) and resided in metropolitan areas (56.8%).

**Table 1:** Participant characteristics

	Participants (N = 227) N (%)
Maternal age	
16–24	77 (33.9%)
25+	150 (66.1%)
Children in care^1^	
1–3	142 (67.6%)
4+	68 (32.4%)
Employment^2^	
Full time	20 (9.5%)
Part time	44 (20.9%)
Unemployed	147 (69.6%)
Residential location	
Metropolitan	129 (56.8%)
Non-metropolitan	98 (43.2%)

^1^N = 210 (data not available for all included participants)

^2^N = 211 (data not available for all included participant)

### Knowledge factors

Knowledge was discussed by participants as critical to ensuring strong oral health practices for their children, with many individuals desiring more education or knowledge in specific areas. Limited nutrition knowledge was a prominent theme across all interviews and all participants. Generally, there were a lot of misconceptions about what is healthy for children, “He does have chocolate but I only give him the Kinder Surprise chocolate because it’s got more of the milk in it.” Further discussion with parents revealed that a lot of this confusion was confounded due to misleading nutrition marketing and resulting nutrition assumptions:

[Baby food is] advertised [as being] good for your baby and healthy for your baby and a lot of them claim … it’s pure fruit, no added sugar… That’s a bit sad because a lot of Mums especially when you’re shopping, you’re busy, you go well this is supposed to be healthy for my baby, it’s on special, I’m going to chuck it in my trolley. And not realising that it could be doing more harm than good.

Many parents cited front of pack marketing as a key information source in terms of nutrition decisions made for their children. When parents were asked to order baby food from highest to lowest according to sugar content, almost all parents who did not read the nutrition label ranked items based on nutrient claims included on product packaging, specifically ‘no added sugar.’ Many carers were taken aback when they discovered that the baby food with the ‘no added sugar’ claim was the highest in sugar: “[That]’s disgusting. They shouldn’t be able to make things like that, they should have a big sign on the front, [with] ‘high sugar content,’ like they do with smoking.”

Limited oral health knowledge concerning topics such as when children should have their first dental visit, when to start using a toothbrush and how much toothpaste is safe for children was common: “Can you brush his teeth too much? Is there a limit to how many times we can brush their teeth during the day?” Many parents were confused or did not have the correct oral health knowledge:

(Interviewer): How would you go about getting the bugs and sugar off his teeth after he's had a bottle and he's sleeping? (Mum): I really don't know. I would just assume that the saliva will wash it away when he's sleeping.

Fluoride knowledge was highly varied, with some parents identifying fluoride as cancer-causing, a whitening agent or a caffeine source. The initial interview for the intervention group occurred during pregnancy and many mothers had misinformation around dental visits during pregnancy, worrying that it could put their baby in harm’s way. Once this was clarified and mothers understood that dental visits were safe, many were willing to go, even if they had not been in years.

Poor parent oral health practices, as a result of limited oral health knowledge, were discussed as a barrier to establishing child oral health. Some parents shared that they cannot expect their children to brush their teeth or reduce sugar consumption when their own actions contrast these expectations. Some parents identified dental visits as low priority because “it’s something I’ve never done” or “I just don’t feel like it.” Other parents discussed oral health as a lower priority amongst competing obligations. This notion provides insight to the practice of only using dental care for emergencies:

I think we’ll probably just [go to the dentist] when he starts to get holes in his teeth or if his teeth are hurting or something fell out or if he’s fallen down and [lost] his tooth and probably then I would take him to the dentist. That’s what I did with all the other ones. When they start to get holes or they need something done to their teeth that’s when I take them to the dentist.

Shock at amount of sugar in baby food, misleading nutrition marketing and nutrition assumptions were the knowledge factors discussed by the highest number of participants; these themes were mentioned most frequently by metropolitan families, families with one to three children and unemployed parents (Additional file 1).

### Parental factors

Parental factors relate to barriers associated with self-identified habits, feelings, or justifications contributing to debilitating oral health habits. The majority of parents identified sugar consumption as detrimental to their child’s oral health, and then utilised concepts including flavour enhancement, sugar cravings, and treating with sugar as justification for exposing their child to sugar. Parents commonly described adding sugar to cereal or water for children to “make it taste better.” Sugar cravings were discussed in terms of parent addiction as well as children; children would often cry or throw a tantrum until parents succumbed to the child’s demands. Some parents justified giving sugar because of their cravings: “You can’t expect them to give up because I’m addicted to Coke, you know, so, just give it.” Using sugar to spoil children was common, even for children who were not yet on solids: “I only give her honey on the dummy every now and then because I like just to give her treats but it’s not all the time.”

Many parents were actively trying to wean children from night-time bottles, more so due to worries about choking or misalignment of teeth, rather than dental decay prevention. Numerous parents talked about caving on bottle removal attempts, often because of the comfort associated with the bottle:

It seems to be her comfort thing for her bed. Like she's got a blanket but... Well she's got a room full of toys too but she seems to like just to lay down and drink a bottle and just play with my hair. And that's how she goes to sleep. So I don't really want to take it away from her because that's her comfort thing.

Convenience of bottles was another barrier to reducing bottle reliance: “It's bad, but there's nothing I can do about it unless I don't want to get any sleep.” A lot of parents were hesitant to wipe their baby’s teeth after feeding because they worried they may disturb the baby’s sleep. Limited time or energy was another barrier; some parents mentioned they had been feeling slack and brushing teeth, making food at home or other preventive behaviours were not their primary concern.

He's very full-on, so [I] just feed him and then do my washing and then after the washing he's probably in something, doing something and it's just too full-on to be able to [read nutrition labels]. If it had on the front of the packaging how many tablespoons of sugar, I'd probably think a second about getting him certain things, but it doesn't. People don't have time to read that. The mums that I know… they just go for what's easy.

The convenience of processed foods and sugar were discussed as a factor of limited time and the easiest option, especially when at sporting or social events and when travelling: “I have tried a few of these [baby foods] when we’re travelling because they made it quite handy to keep in the esky and just whip it out to give her something.” For parents who identified low water consumption in their children, the two primary barriers were child aversion to water and low household tap water use. Some households used rainwater as their primary source of water usually due to access and taste preference.

Limited time or energy, exposure to sugar and comfort of bottle were the parental barriers discussed by the highest number of participants; these three themes were cited most frequently by families in non-metropolitan areas, families with one to three children, unemployed parents, and older parents. (Additional file 1).

### Structural factors

Physical distance from dental providers as well as long wait times were structural barriers for families, with mothers waiting between eighteen months and eight years for a public dentist appointment. Many participants discussed a fear of dental visits as a barrier to booking and attending appointments. Several parents were under the impression that school dental visits, common in primary schools across Australia, were sufficient in place of regular dental check-ups. This assumption prevented parents from taking their children for dental visits and waiting until the child was at least 5 years old and eligible for the school programs. While this program intends to facilitate strong oral health, it created confusion for participants around when to access dental services for their children:

In kindy they take [kids] to the dentist and stuff. I just thought when they go to kindy they usually send home a note saying there’s a dentist coming, is it alright if they can see you? The dentist comes and if the dentist says there’s any problems that’s when I’ll take them to the dentist. I never thought about taking her before… I thought all kids just went to the dentist when they went to school.

Financial limitations were discussed in terms of the cost of dental care and transportation to appointments. When participants were informed about funding schemes or offered transportation to dental visits, many that had previously been unable to go were happy to attend. Parents cited previous negative healthcare experiences as a barrier to pursuing preventive healthcare and these experiences directly influenced parental perceived negative reaction of children at their first dental visit. Specific stories of negative dental experiences were shared, and a few parents discussed experiences of racism:

I’ve noticed with when you go to doctors and all that … like dentists, especially being a black woman they don’t talk to you, they talk to the secretary, or whatever it is, about you. And then you’ve got to remind them hello, I’m sitting in the room you know, you’ve got to kind of put your foot down… I think that they think that I don’t know what … they’re talking about, you know… I want to be treated with the proper respect that everybody else gets because you can see it when you walk into the doctors they look at you like oh, another black person.

The structural component of sugar consumption related to the sheer availability of sugar. The industrialisation of food production has rapidly transformed the food landscape for communities, especially for those in rural and remote areas, where reliance on processed foods have increased due to limited access to fresh foods. Parents expressed being overwhelmed at the availability of sugar and exhaustion at navigating which foods are healthy for their families: “It's terrible. It’s just in everything, sugar’s in everything. And like I said, you know, some things you think there's not much sugar in them, [but] it's you know right at the top [of the ingredient list].”

Parent perceived negative reaction of child at dental visits, availability of sugar and financial limitations were the structural barriers cited by the greatest number of families, lack of transportation was only mentioned as a barrier by unemployed parents and experiences of racism when accessing health services was only mentioned by employed parents (Additional file 1).

### Social factors

Social factors were concerned with community and social environments in which oral health exists. Limited family support was a barrier for parents who needed help with transportation or babysitting for dental visits. Some parents touched on the difficulty of maintaining oral health when extended family assist with childcare but do not respect their routines. Parents with limited partner support described the burden of responsibility for all aspects of their children’s lives, which often resulted in a lowered priority for oral health. Limited partner support was also discussed as poor communication or respect between parents regarding oral health routines. Lack of oral health social discussions at parent support groups or among friends was commonly discussed.

Household role models, including parent and sibling negative influence, were identified as barriers to establishing oral health practices for children. Parents identified themselves as a negative influence with regard to sugar consumption, saying that they couldn’t justify limiting their children’s sugar when they were addicted. Many parents described letting older children have sugar, which frequently resulted in the taunting of younger child or sneaking them lollies. Sugar consumption was heavily impacted by social settings and special occasions. Family members giving sugar was the most common theme within social factors; parents expressed frustration at family members disobeying their wishes and overriding their efforts to restrict sugar consumption. Parents suggested that giving sugar was a way for relatives to show their love, but many families dismissed the potential impacts on the child’s health. Some parents even talked about staying at home more often to control sugar consumption: “When we go to my mum and dad’s it’s like because my other nephew is there and they’ve got [sugar] … and baby [goes], what’s that? So that’s why we try and stay home.”

Family members giving sugar was the social barrier cited by the highest number of participants, across all subgroup characteristics. Younger parents and those in non-metropolitan regions frequently discussed the barrier of negative sibling and parental role model. Families in metropolitan regions most frequently discussed sugar on special occasions and a lack of social oral health discussions (Additional file 1).

## Discussion

Indigenous oral health inequities in Australia are well documented [4, 15, 16, 18, 22, 47–49]. However, few projects have highlighted Indigenous voices and documented personal perspectives, providing context for the experience of Indigenous oral health in Australia [27, 50–53]. This project is unique in that it employed an open-ended approach to discussion, through the use of MI, and provided space for participants to ask questions and direct the conversation [30]. The results emphasise the multi-faceted circumstances in which Indigenous oral health exists for new mothers and their children – with identification of barriers across parental, structural, social and knowledge factors. Many findings from this project reinforce previously identified barriers to oral health for Indigenous communities including: availability of sugar [18, 51], inaccessibility of oral health care [15, 27, 54], racism [27, 52, 55], poor parent oral health practices [53, 56, 57], lack of accessible transport [27, 52, 57], limited time and energy [50], competing health priorities [27, 50, 51], waiting times [27, 52], financial limitations [27, 50–52, 55, 58], school dental programs [50, 52], limited oral health knowledge [27, 53, 54] and limited nutrition knowledge [51, 52].

Findings of self-identified poor parent oral health practices, fear of dentist, waiting lists, physical distance from dentist, financial limitations, negative health care experiences and limited oral health knowledge work together to tell an important story that parents shared during this project. The impact of these factors results in low dental attendance and lack of emphasis on prevention, which is alarming because regular dental visits increase the probability of diagnosing, managing and limiting oral disease [59]. Similarly, Butten et al. [51] found a lack of prevention efforts amongst Indigenous mothers in Queensland due to the complex interplay of financial, personal and structural factors. The availability of school dental programs shaped parents’ perceptions of child oral health needs in this project, which limited prevention efforts as parents did not identify a need to take children for dental visits earlier; for many five-year-old children, it is too late for preventive actions and restorations are needed. Indigenous mothers in Queensland utilised school dental programs for older children, but many did not take their pre-school children for dental visits [50]. Regular dental attendance and prevention efforts underscore healthy trajectories, behaviours and improved quality of life for children [59–61]. Indigenous children have the highest rates of dental surgery under general anaesthesia and the occurrence is increasing; in Australia, Indigenous children have twice the rate of hospital-based dental surgery under a general anaesthetic compared to non-Indigenous children [47]. The high cost, risks and logistical implications of dental surgery, as well as recurrence of disease provides precedence for the prioritisation of prevention over treatment of ECC [13]. Additionally, prevention is the most cost-effective mechanism to addressing ECC, with research suggesting that fifty dollars is saved on restoration procedures for every dollar spent on prevention [62]. Findings from this project are representative of carers with children 36 months and younger; Indigenous mothers from Queensland have described the increased difficulties experienced when trying to maintain oral health routines as children age, which further stresses the importance of establishing good oral health habits at a young age [50].

Limited parental oral health knowledge impacts a child’s oral health due to the close relationship between caregiver oral health and child oral health [18]. Both parent tooth brushing habits and attitudes or knowledge towards oral health have been associated with ECC development in children [63, 64]. Limited oral health knowledge directly impacts child health as poor maternal oral health is related to adverse birth outcomes [65]. This trial encouraged pregnant mothers to attend dental appointments and to take children around 18 months of age. Limited oral health knowledge persisted among participants who attended dental appointments, highlighting the importance of sustained awareness efforts and behaviour change programs for oral health prevention. Dental services are not covered for adults under Medicare, the public funding system, in Australia; while a public dental service exists, they are stipulated by eligibility criteria and often have long wait times and private options require large out-of-pocket fees [66]. Our findings highlighted common misinformation around dental visits during pregnancy. Current evidence-based guidelines recommend that women seek dental care early in pregnancy and identify the importance of midwives in facilitating this, however access for many pregnant mothers remains low [52, 67]. Previous research suggests that lack of referral knowledge and competing health matters are barriers to prioritising oral health for midwives in Australia [68] and limited oral health training exists for Aboriginal Health Workers [27]. Mandating oral health education for all health professionals has been suggested as a way to increase accessibility of oral health [50]. The importance of culturally appropriate, ongoing and informal dissemination of oral health information has been noted elsewhere [52, 54] and the lack of social oral health discussions identified by participants in this project reinforces the need for community-level education and health promotion.

Knowledge factors also extended to nutrition knowledge in our project. Misleading nutrition marketing, nutrition assumptions, shock at amount of sugar in baby food and not reading nutrition labels were all findings related to limited nutrition knowledge. Limited knowledge in conjunction with other factors such as limited time or energy, convenience of processed foods, financial limitations and availability of sugar, resulted in a constrained ability of parents to make healthy food choices. For the majority of parents in our project, nutrient claims were the primary source of nutrition information and largely influenced food decisions. Many parents were upset once they realised the nutrition assumptions that they had made due to misleading marketing. Similarly, Indigenous mothers in Queensland identified that they had ‘done the wrong thing’ by giving children milk or juice because they believed it was healthy. The concept of a ‘health halo’ has been reported in previous research and occurs when nutrient or health claims lead to consumer interpretations of a product being healthier than it actually is [69]. In our project, one parent suggested using warning labels for high sugar content, similar to cigarette packaging; a First Nations participant in a Canadian study suggested the same idea: “…[T]he same scope of thinking [like] they do with cigarettes: they should put [warning labels] on the candy bars” [54]. The relationship between these factors underscores the importance of nutrition education for parents and consideration of the impact that nutrient claims on baby foods have on nutrition assumptions and food choices. Stronger regulation for claims using nutrient profiling has previously been called for in Australia due to consumer tendency to infer health benefits as highlighted in our findings [70].

In this study, sugar consumption included was related to availability, social influences, limited parental nutrition knowledge, flavour enhancement and convenience of sugary foods and drinks. It is well understood that dietary factors, specifically sugar consumption, increase the availability of fermentable carbohydrates required for acid formation and ECC development, while simultaneously increasing host susceptibility due to the influence of prenatal and infant nutrition on enamel development [56, 71]. The misconception that baby teeth are less important than permanent dentition was cited as rationale for exposing children to sugar, this perception has previously been identified as a barrier to preventive care in young Indigenous children [50]. Many parents in our project talked about the availability of sugar as a barrier to decision making because “it’s everywhere.” Similarly, Indigenous mothers from Queensland identified lower sugar exposure during their own childhood, when compared to their children, because processed foods were not as common [50]. The transition from traditional diets to Western diets, due to processes of colonisation and a loss of traditional foods, has been explored and identified as a contributing factor to a variety of health inequities experienced by Indigenous peoples globally [7]. Sugar consumption is influenced by many factors within the home, school and wider community environments [18, 72]. Even when parents are attempting to limit consumption, they cannot control what happens in schools or with other family members. Beyond an increase in knowledge, healthy food choices need to be possible within a given environment and when education efforts do not consider environmental influence, they are ineffective at initiating behaviour change [73].

The subgroup comparison provided insight into how different socioeconomic positions influence barriers to establishing oral health practices for Indigenous children. Lower socioeconomic status is directly related to oral health disparities in Australia [68] and, indeed, globally [74]. One of the largest trends was that families living in non-metropolitan areas were more likely to identify barriers across all subgroups than those living in metropolitan areas. This finding highlights the need for holistic, targeted dental public health efforts in rural and remote communities across South Australia. Parent employment status, children in care and maternal age impacted frequency of barrier identification in various ways. The subgroup comparison highlights barriers for families of different demographics and has the potential to inform future policy, research and interventions for specific subsets of the population.

### Strengths and limitations

This paper adds to the limited qualitative research on Indigenous oral health in Australia and highlights Indigenous voices that illustrate the challenges carers face in optimising oral health for their children within Westernised environments. The structural barriers identified by parents are part of a system that has historically excluded Indigenous voices despite their direct impact on Indigenous health [75]. A strength of this project is the use of MI as the conversational methodology respected through Indigenous traditions of yarning, and provided the space and time for participants to engage in conversations [30]. The variation in prominence of themes is representative of participant’s experiences due to the structure of MI where the interviewer is positioned as a knowledgeable person accessible for participants to engage with on topics, rather than prioritising topics and questions with a structured guide. Additionally, this project is unique in that socioeconomic positions were compared to identify how barriers exist for families in different situations. A limitation of the study is that baseline measures for age and employment used for subgroup comparisons reflect maternal characteristics rather than the entire household. Additionally, the majority of interviews were conducted with mothers at baseline occurred during pregnancy, however future projects would be more impactful by prioritising paternal participation and engaging the whole family, as aligned with cultural understandings of holistic health.

## Conclusion

Despite the barriers shared by participants and discussed here, parents understood the importance of oral health and desired the best possible outcome for their children’s teeth. Policymakers, researchers and public health professionals are urged to consider the barriers experienced firsthand by Indigenous peoples and prioritise Indigenous partnerships when addressing oral health disparities. Our recommendations from these findings include an increased focus on oral health promotion efforts in non-metropolitan areas; the utilisation of community experiences and needs in creating useful strategies that encourage oral health and nutrition knowledge; and the extension of oral health initiatives and future research to include all family members.

## Supplementary Information

**Additional file 1.** Participant comparison by demographic characteristics within knowledge, parental, structural, and social factors.

## Data Availability

The datasets analysed during the current study are not publicly available due to confidentiality concerns but are available from the corresponding author on reasonable request.

## Abbreviations

ECC: Early Childhood Caries
FT: Full-time
Metro: Metropolitan
Non-metro: Non-Metropolitan
MI: Motivational Interviewing
PT: Part-time
UE: Unemployed
