# Supplementary material for: “What are we doing to our babies’ teeth?” Barriers to establishing oral health practices for Indigenous children in South Australia
Source: BMC Oral Health. 2021 Sep 6;21:434. doi: 10.1186/s12903-021-01791-x (PMC8422744; doi:10.1186/s12903-021-01791-x)
Supplement: Supplementary file 1 — Additional file 1. Participant comparison by demographic characteristics within knowledge, parental, structural, and social factors. [file 12903_2021_1791_MOESM1_ESM.docx]

Supplementary File 1

STable 1. Participant comparison by demographic characteristics within knowledge factors.

|  |  | Residency | | Children in care | | Employment^2^ | | | Age | |
| --- | --- | --- | --- | --- | --- | --- | --- | --- | --- | --- |
| Knowledge Factors | TotaL^1^ | Metro | non-metro | 1 = 1-3 | 2 = 4+ | FT | PT | UE | 14-24 | 25+ |
| Shock at sugar in baby products | 152 | 65 | 87 | 101 | 40 | 13 | 31 | 97 | 52 | 100 |
| Nutrition assumption | 106 | 49 | 57 | 74 | 24 | 8 | 21 | 69 | 39 | 67 |
| Misleading nutrition marketing | 104 | 44 | 60 | 67 | 29 | 9 | 23 | 64 | 38 | 66 |
| Misinformation around dentist and pregnancy | 91 | 32 | 59 | 59 | 25 | 7 | 20 | 57 | 31 | 60 |
| Limited oral health knowledge | 83 | 25 | 58 | 60 | 26 | 8 | 22 | 56 | 27 | 66 |
| Limited nutrition knowledge | 66 | 24 | 42 | 43 | 19 | 7 | 12 | 43 | 21 | 45 |
| Not reading nutrition labels | 41 | 21 | 20 | 26 | 13 | 1 | 10 | 28 | 12 | 29 |
| Limited fluoride knowledge | 23 | 9 | 14 | 19 | 2 | 1 | 5 | 15 | 9 | 14 |
| Poor parent oral health practices | 19 | 5 | 14 | 14 | 4 | 3 | 2 | 13 | 8 | 11 |
| Dentist only for emergency | 17 | 5 | 12 | 7 | 7 | 1 | 1 | 12 | 9 | 8 |
| Dental visits low priority | 10 | 1 | 9 | 9 | 1 | 1 | 1 | 8 | 5 | 5 |

^1^Total number of participants that discussed each theme *Note* demographic data for children in care and employment was not available for all participants

^2^FT = full-time, PT = part-time, UE = unemployed

STable 2. Participant comparison by demographic characteristics within parental factors.

|  |  | Residency | | Children in care | | Employment^2^ | | | Age | |
| --- | --- | --- | --- | --- | --- | --- | --- | --- | --- | --- |
| parental Factors | TOTAL^1^ | Metro | non-metro | 1-3 | 4+ | FT | PT | UE | 14-24 | 25+ |
| Limited time or energy | 79 | 35 | 44 | 48 | 23 | 6 | 22 | 43 | 25 | 54 |
| Exposure to sugar | 72 | 20 | 52 | 40 | 27 | 5 | 10 | 52 | 21 | 51 |
| Comfort of bottle | 58 | 23 | 35 | 39 | 13 | 8 | 13 | 31 | 16 | 42 |
| Sugar cravings | 48 | 18 | 30 | 27 | 18 | 3 | 13 | 29 | 14 | 34 |
| Convenience of processed foods | 46 | 27 | 19 | 34 | 9 | 5 | 13 | 25 | 14 | 32 |
| Treat with sugar | 32 | 13 | 19 | 19 | 10 | 2 | 4 | 23 | 14 | 18 |
| Caving on bottle | 31 | 10 | 21 | 19 | 10 | 5 | 4 | 20 | 9 | 22 |
| Convenience of sugar | 25 | 9 | 16 | 18 | 4 | 1 | 7 | 14 | 4 | 21 |
| Child aversion to water | 18 | 9 | 9 | 9 | 8 | 2 | 7 | 8 | 6 | 12 |
| Convenience of bottle | 16 | 8 | 8 | 10 | 5 | 1 | 3 | 11 | 4 | 12 |
| Disturbing baby’s sleep with wiping | 16 | 4 | 12 | 13 | 2 | 1 | 4 | 10 | 7 | 9 |
| Enhancing flavour | 12 | 5 | 7 | 5 | 7 | 0 | 2 | 10 | 5 | 7 |
| Low tap water use | 11 | 2 | 9 | 8 | 1 | 1 | 2 | 6 | 3 | 8 |
| Parent feeling slack | 9 | 5 | 4 | 5 | 4 | 1 | 1 | 7 | 4 | 5 |

^1^Total number of participants that discussed each theme *Note* demographic data for children in care and employment was not available for all participants

^2^FT = full-time, PT = part-time, UE = unemployed

STable 3. Participant comparison by demographic characteristics within structural factors.

|  |  | residency | | Children in care | | Employment^2^ | | | Age | |
| --- | --- | --- | --- | --- | --- | --- | --- | --- | --- | --- |
| Structural Factors | TOTAL^1^ | Metro | non-metro | 1-3 | 4+ | FT | PT | UE | 14-24 | 25+ |
| Parent perceived negative child reaction at dentist | 71 | 30 | 41 | 46 | 21 | 8 | 17 | 42 | 24 | 47 |
| Availability of sugar | 38 | 16 | 22 | 22 | 13 | 2 | 6 | 27 | 12 | 26 |
| Financial limitations | 38 | 15 | 23 | 23 | 12 | 3 | 6 | 26 | 10 | 28 |
| Cost of dentist | 34 | 13 | 21 | 21 | 11 | 1 | 8 | 23 | 16 | 18 |
| Fear of dentist | 25 | 7 | 18 | 13 | 12 | 2 | 2 | 21 | 10 | 15 |
| School programs as a barrier | 17 | 8 | 9 | 11 | 4 | 1 | 5 | 9 | 11 | 6 |
| Waiting list | 16 | 7 | 9 | 12 | 4 | 2 | 3 | 11 | 5 | 11 |
| Negative dental experiences | 13 | 3 | 10 | 5 | 7 | 2 | 1 | 9 | 4 | 9 |
| Negative health care experiences | 11 | 3 | 8 | 6 | 4 | 2 | 1 | 7 | 4 | 7 |
| Physical distance from dentist | 10 | 3 | 7 | 5 | 5 | 1 | 2 | 7 | 0 | 10 |
| Lack of transportation | 7 | 2 | 5 | 2 | 5 | 0 | 0 | 7 | 1 | 6 |

^1^Total number of participants that discussed each theme *Note* demographic data for children in care and employment was not available for all participants

^2^FT = full-time, PT = part-time, UE = unemployed

STable 4. Participant comparison by demographic characteristics within social factors.

|  |  | residency | | ChildREn in care | | Employment^2^ | | | Age | |
| --- | --- | --- | --- | --- | --- | --- | --- | --- | --- | --- |
| Social Factors | Total^1^ | Metro | non-metro | 1-3 | 4+ | FT | PT | UE | 14-24 | 25+ |
| Family members giving sugar | 71^2^ | 28 | 43 | 52 | 15 | 5 | 17 | 45 | 52 | 15 |
| Social influences | 23 | 10 | 13 | 15 | 6 | 4 | 4 | 14 | 15 | 6 |
| Sugar on special occasions | 22 | 12 | 10 | 15 | 6 | 2 | 5 | 14 | 15 | 6 |
| Lack of oral health social discussions | 22 | 11 | 11 | 16 | 5 | 1 | 6 | 14 | 16 | 5 |
| Limited partner support | 22 | 10 | 12 | 14 | 7 | 2 | 6 | 13 | 14 | 7 |
| Parent as negative role model | 20 | 6 | 14 | 13 | 7 | 2 | 5 | 13 | 13 | 7 |
| Sibling as negative role model | 18 | 3 | 15 | 10 | 7 | 2 | 3 | 12 | 10 | 7 |
| Limited family support | 16 | 9 | 7 | 12 | 4 | 0 | 4 | 12 | 12 | 4 |

^1^Total number of participants that discussed each theme *Note* demographic data for children in care and employment was not available for all participants

^2^FT = full-time, PT = part-time, UE = unemployed
